# The Role of Farnesoid X Receptor in Accelerated Liver Regeneration in Rats Subjected to ALPPS

**DOI:** 10.3390/curroncol28060438

**Published:** 2021-12-09

**Authors:** Noemi Daradics, Pim B. Olthof, Andras Budai, Michal Heger, Thomas M. van Gulik, Andras Fulop, Attila Szijarto

**Affiliations:** 1 Hepato-Pancreatico-Biliary (HPB) Surgical Research Center Hungary, Department of Surgery, Transplantation and Interventional Gastroenterology, Semmelweis University, 1082 Budapest, Hungary; fulop.andras2@gmail.com (A.F.); szijartoattila@gmail.com (A.S.); 2 Erasmus Medical Center, Department of Surgery, 3015 GD Rotterdam, The Netherlands; p.b.olthof@amsterdamumc.nl; 3 2nd Department of Pathology, Semmelweis University, 1091 Budapest, Hungary; andras.budai.md@gmail.com; 4 Jiaxing Key Laboratory for Photonanomedicine and Experimental Therapeutics, Department of Pharmaceutics, College of Medicine, Jiaxing University, Jiaxing 314001, China; M.Heger@uu.nl; 5 Laboratory of Experimental Oncology, Department of Pathology, Erasmus Medical Center, 3015 GD Rotterdam, The Netherlands; 6 Department of Surgery, Academic Medical Center, 1105 AZ Amsterdam, The Netherlands; t.m.vangulik@amsterdamumc.nl

**Keywords:** surgical oncology, liver regeneration, ALPPS, Fxr, bile acid, in vivo research

## Abstract

Background: the role of bile acid (BA)-induced farnesoid X receptor (Fxr) signaling in liver regeneration following associating liver partition and portal vein ligation for staged hepatectomy (ALPPS) was investigated in a rat model. Methods: Male Wistar rats underwent portal vein ligation (PVL) (*n* = 30) or ALPPS (*n* = 30). Animals were sacrificed pre-operatively and at 24, 48, 72, or 168 h after intervention. Regeneration rate, Ki67 index, hemodynamic changes in the hepatic circulation, and BA levels were assessed. Transcriptome analysis of molecular regulators involved in the Fxr signaling pathway, BA transport, and BA production was performed. Results: ALLPS induced more extensive liver regeneration (*p* < 0.001) and elevation of systemic and portal BA levels (*p* < 0.05) than PVL. The mRNA levels of proteins participating in hepatic Fxr signaling were comparable between the intervention groups. More profound activation of the intestinal Fxr pathway was observed 24 h after ALPPS compared to PVL. Conclusion: Our study elaborates on a possible linkage between BA-induced Fxr signaling and accelerated liver regeneration induced by ALPPS in rats. ALPPS could trigger liver regeneration via intestinal Fxr signaling cascades instead of hepatic Fxr signaling, thereby deviating from the mechanism of BA-mediated regeneration following one-stage hepatectomy.

## 1. Introduction

Liver regeneration is fundamental to successful and safe liver resections. Approximately 45% of patients with advanced tumors undergo extensive partial hepatectomy (PHx) with curative intent [1]. After major PHx, an insufficient volume and functioning of the liver remnant can cause life-threatening post-hepatectomy liver failure [2]. Accordingly, liver regeneration and the enhancement of the future liver remnant (FLR) are key processes in the clinical management of hepatic malignancies.

Portal vein occlusion techniques such as portal vein embolization (PVE) and portal vein ligation (PVL) are standardly employed before PHx to pre-operatively increase FLR size [3]. These techniques come with drawbacks. Firstly, the growth of functional liver volume after PVE and PVL requires 4–8 weeks, which could lead to tumor spread and progression and thereby compromise the planned PHx [4]. Secondly, the extent of FLR growth induced by PVE and PVL is not always sufficient to perform a safe PHx. A novel method called associating liver partition and portal vein ligation for staged hepatectomy (ALPPS) can overcome these hurdles, as ALPPS triggers rapid and more robust liver regeneration (FLR growth of 40–150% versus 20–50% after PVL) within 6–10 days [5].

The initiation, propagation, and termination of accelerated liver regeneration following ALPPS are orchestrated by a series of intricate signals and pathways [6,7], although the exact mechanisms are still not fully understood. Bile acids (BAs) occupy a central role in the early phase of liver regeneration [8] and it is therefore plausible that BAs also mediate liver regeneration after ALPPS. Certain BAs have mitotic properties and act as signaling molecules that bind and activate the farnesoid X receptor (Fxr) in the nucleus. Fxr activation by BAs subsequently triggers hepatocyte proliferation [9]. PHx leads to BA overload in the remaining hepatocytes that in turn drives the regenerative response [10,11,12]. Disruption of bile flow delays liver regrowth, possibly due to hampered Fxr signaling, which is essential for normal liver regeneration. Corroboratively, *Fxr*-deficient mice exhibit significantly impaired liver regeneration following hepatectomy [9,13].

**Figure 1:**
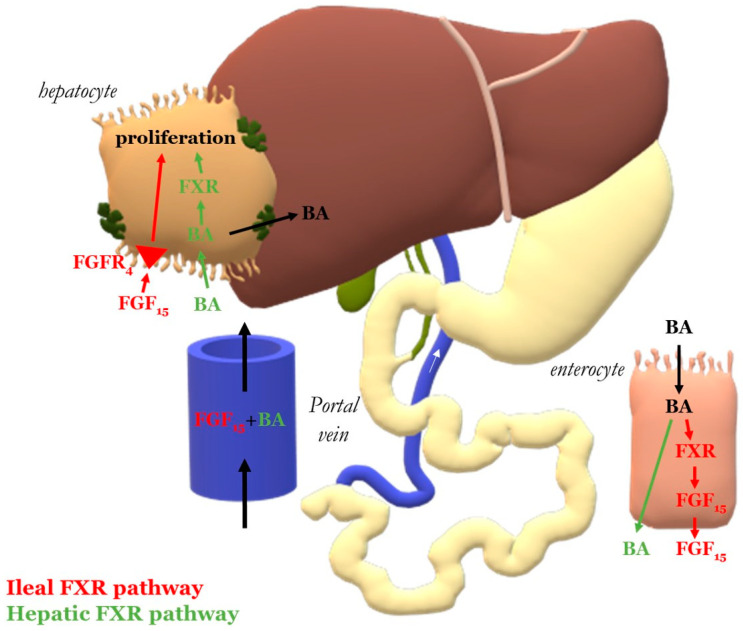
Bile acid (BA) and farnesoid X receptor (Fxr) signaling. Ileal Fxr pathway (red marking): The absorbed BAs activate Fxr signaling in the ileal enterocytes, inducing the production of mitogenic fibroblast growth factor (Fgf)15. After entering the portal circulation, Fgf15 binds to hepatocytes and activates the regeneration pathway via the Fgfr4 receptor. Hepatic Fxr pathway (green marking): absorbed BAs enter the portal circulation and reach the hepatocytes, where they activate hepatocyte Fxr signaling and initiate proliferation.

Fxr is expressed both in hepatocytes and enterocytes and steers a hepatic and intestinal route of liver regeneration (Figure 1). As the deletion of hepatic *Fxr* does not completely inhibit but only stalls liver regeneration [13], the intestinal Fxr signaling pathway might be the primary activator of liver regeneration through the production of mitogenic fibroblast growth factor (Fgf)19 (Fgf15 ortholog in rodents) [14]. Fgf15 regulates BA synthesis and activates liver regeneration via the hepatocyte Fgfr4 receptor. The critical role of Fgf15 in the regenerative process is evidenced by the significant impairment of liver regrowth in Fgf15-deficient mice [15].

The role of Fxr signaling in liver regeneration following PVL has already been investigated [16,17,18], although the importance of the hepatic and intestinal pathways has not yet been fully elucidated. Currently, there is no data regarding Fxr signaling following ALPPS. The function of BAs in liver regeneration and the involvement of hepatic and intestinal Fxr signaling in this process were therefore investigated in rats subjected to ALPPS and juxtaposed to PVL. It was hypothesized that ALPPS would cause an increase in the plasma levels of BAs that would activate the Fxr-Foxm1b signaling axis in the hepatocytes comprising the FLR, translating to liver growth. Moreover, it was expected that the excessive BAs that had been taken up by liver cells would be excreted into the bile to restore intracellular BA homeostasis, leading to their reuptake by enterocytes and BA-induced ileal Fxr signaling. The ileal Fxr signaling would subsequently give rise to the upregulation of Fgf15, which in turn would be excreted into the enterohepatic circulation and bind to its cognate receptor Fgfr4 on hepatocytes, leading to cell proliferative signal amplification via the Fxr-Foxm1b pathway, among others, and eventually accelerated liver growth compared to PVL alone. The present study focused on several key aspects of this hypothesis.

## 2. Methods

All experiments were reviewed and approved by the scientific and ethics board of animal experimentation of the National Department of Food Chain Safety (approval number: PE/EA/1843-6/2016) and reported according to the ARRIVE criteria [19].

### 2.1. Animals and Operative Procedures

Male Wistar rats (Toxicoop, Budapest, Hungary), weighing between 180 and 220 g, were housed in a humidity- (40–70%) and temperature- (20–22 °C) controlled environment with a 12 h day–night cycle and ad libitum access to standard chow (Toxicoop) and water. Rats were acclimated for 7 days before inclusion in the experiments.

**Figure 2:**
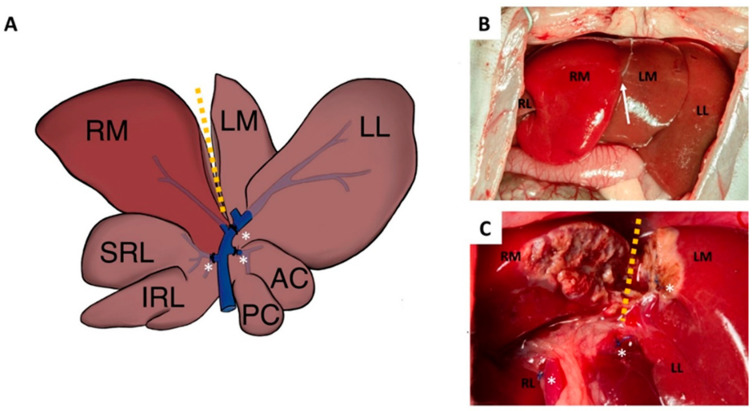
Schematic figure (**A**) and photographs (**B**,**C**) of the operative procedure on rats. Following median laparotomy, in the case of portal vein ligation (PVL), the portal branches leading to the right lateral (RL), left part of the median lobe (LM), left lateral lobe (LL), and the caudate lobe were ligated (*). The non-ligated lobe comprised the right median (RM) lobe. In the case of ALPPS, additional transection (yellow dotted line) was performed alongside the transition line (white arrow) between the RM and LM lobes.

Following induction of anesthesia with isoflurane (2–2.5% *v*/*v*; 1 L/min air), rats underwent either PVL (*n* = 30) or ALPPS (*n* = 30), which were performed as described previously [20]. Briefly, during PVL, ligation of portal branches leading to the right lateral, left part of the median, left lateral, and caudate lobes were performed (70% of the liver). In the case of ALPPS, additional transection was performed alongside the transition line between the left and right part of the median lobe (Figure 2). Liver wounds were sealed carefully by electrocauterization. Animals received antibiotic treatment (10 mg/kg body weight metronidazole intraperitoneally) and analgesia (1 mg/kg nalbuphine subcutaneously, repeated once 24 h post-operatively).

### 2.2. Sample Extraction

The animals were sacrificed by exsanguination via cardiac puncture after intraperitoneal injection of 75 mg/kg ketamine and 7.5 mg/kg xylazine in 1.5 mL saline solution. Animal sacrifice was performed at baseline (without any intervention) and 24, 48, 72, or 168 h following PVL and ALPPS (*n* = 6 per group). Before exsanguination the body weight of each animal was determined (model SCL-1053, Kent Scientific, Torrington, CT, USA).

Portal blood was collected into heparinized tubes (Vacutainer) directly before cardiac puncture from the portal vein using a 24 G needle. The remainder of the systemic blood was collected into heparinized tubes by cardiac puncture. The liver and a standard part of the ileum were extracted after sacrifice. Approximately 150 mg tissue from the right median lobe was snap frozen in liquid nitrogen and another 150 mg was fixed in 4% buffered formaldehyde for histology. Right median lobe samples were snap frozen in liquid nitrogen and stored at −80 °C until further use.

### 2.3. Quantification of Liver Regeneration

Each liver lobe was measured separately using an analytical scale (model AG245, Mettler Toledo, Columbus, OH, USA). Percentual increase in liver mass was calculated using the following formula: (lobe weight/body weight at the time of death)/(mean lobe weight at preoperative time point/ body weight at preoperative time point) × 100%. For the right lateral, left lateral, caudate, and left part of the median lobe the outcome was atrophy, from which we calculated the liver mass of the ligated lobes during the experiment by the following formula: (lobe weight of ligated lobes/body weight at the time of death)/(mean lobe weight of ligated lobes/body weight at preoperative time point) × 100%.

### 2.4. Histology

From the formalin-fixed paraffin-embedded (FFPE) liver tissue specimens, 4-µm thick sections were cut, deparaffinized in xylene (2 × 10 min), and rehydrated in a graded alcohol series. For routine structural analysis of the liver hematoxylin and eosin stain was used. Antigen retrieval was performed at pH = 6.0 (S2031, Agilent, Santa Clara, CA, USA). Ki67 immunohistochemistry was performed using anti-Ki67 antibodies (ab16667, Abcam, Cambridge, UK) according to the manufacturer’s instructions. Counterstaining was performed with hematoxylin. The histological slides were scanned with a Pannoramic P1000 slide scanner system (3DHistech, Budapest, Hungary) and analyzed with QuPath software [21]. The Ki67 index was calculated on the whole slide as the number of Ki67-positive cells per total number of cells.

### 2.5. Assessment of Hepatic Microcirculation

The microcirculation of the liver was evaluated by laser Doppler flowmetry (LDF) (Moor Instruments, London, UK) during the surgical steps: 5 min after opening of the abdomen (baseline) and 5 min after the ligation of portal branches (in the case of PVL) or the transection (in the case of ALPPS). Individual measurements were performed for 1 min with a surface probe positioned at 3 sites on the right part as well as the left part of the median lobe. A mean value was calculated.

### 2.6. Assessment of Portal Pressure

The right portal branch was cannulated with a 24 G needle before sacrifice and an invasive blood pressure monitoring device (model DRT4-3109, Kent Scientific, Torrington, CT, USA) was connected to register portal pressure for 5 min at fixed needle position. Data were recorded with DasyLab software (v9.00.02, National Instruments, Austin, TX, USA).

### 2.7. Quantification of Total Bile Acid Concentration

The total BA concentration was determined in heparin-anticoagulated plasma samples with the Total Bile Acids Assay Kit from Diazyme (Poway, CA, USA) using a slightly modified version of the kit manual. In brief, 135 µL of >0.1 mM Thio-NAD (‘Reagent 1′) was added to 4 µL of sample, standard (50 µM conjugated cholic acids) or blank (NaCl), and incubated for 5 min at 37 °C in the dark. Subsequently, 45 µL of 2 kU/L 3-α-HSD/>0.1 mM NADH (‘Reagent 2′) was added to each well, after which the absorption at 405 nm was read at 2 min intervals during 12 min on a Synergy HT microplate reader (Biotek, Winooski, VT, USA) at 37 °C. The BA concentration was calculated as per manufacturer’s instructions. For samples with high BA concentrations, only the data points of the kinetic read over in which absorption increased linearly (i.e., with an R^2^ of > 0.99) were used for data analysis.

### 2.8. Quantitative Real-Time Polymerase Chain Reaction (qRT-PCR)

A quantity of 35 mg of tissue was homogenized in 150 μL of ice-cold phosphate-buffered saline by using a MagNA Lyser (Roche Applied Sciences, Basel, Switzerland). RNA was isolated with an RNeasy Mini Kit (Qiagen, Hilden, Germany) and quantified with a NanoDrop (Thermo Fisher Scientific, Waltham, MA, USA). Reverse transcription of 1 μg of mRNA to cDNA was performed using a SensiFAST cDNA Synthesis Kit (Bioline, London, UK) according to the manufacturer’s instructions. qRT-PCR was conducted on a LightCycler 480 (Roche) using the SensiFAST SYBR No-ROX mix (Bioline). LinReg software was used to analyze the fluorescence traces [22]. Transcript levels of the genes of interest were normalized to those of the housekeeping genes hypoxanthine phosphoribosyltransferase 1 (*Hprt1*) for ileum samples and the geometric mean of beta-2-microglobulin (*B2m*) for liver samples. These housekeeping genes proved to be most stable. Primer sequences spanning an intron–exon or exon–exon junction were derived in an NCBI Primer Blast. The genes of interest and their forward and reverse primer sequences are listed in Supplementary Table S1. Primer specificity was validated by melting curve analysis and agarose gel electrophoresis.

For purposes of simplicity, gene names were denoted by their protein name in italics.

### 2.9. Statistical Analysis

Statistical analysis was performed in GraphPad Prism (GraphPad Software, La Jolla, CA, USA). Data were analyzed with a two-way ANOVA with Tukey’s test for post hoc analysis on the basis of a normally distributed data set and homocedasticity. A *p*-value of ≤0.05 was considered statistically significant. To evaluate the model fit and our assumptions of the data, we employed diagnostic tests before performing a two-way ANOVA.

## 3. Results

### 3.1. Accelerated Liver Growth and Cell Proliferation following ALPPS

**Figure 3:**
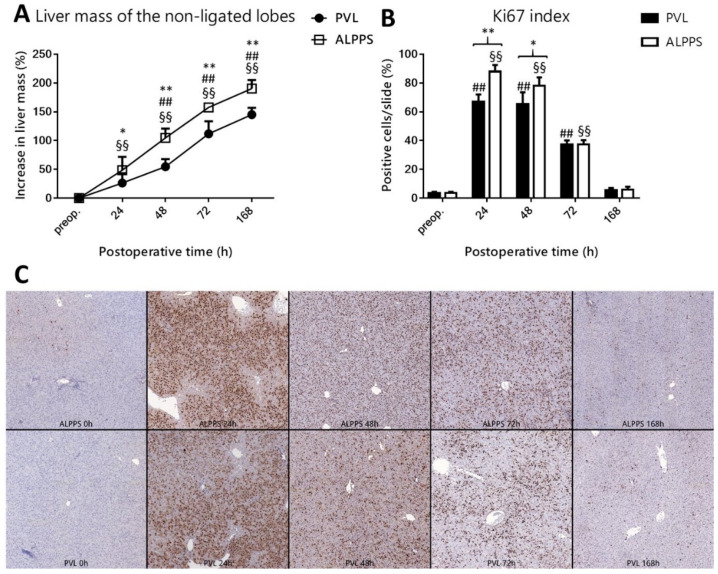
Effect of portal vein ligation (PVL) and associating liver partition and portal vein ligation for staged hepatectomy (ALPPS) on right medial lobe mass and hepatocellular proliferation. Increase in liver mass of non-ligated lobe (**A**) and Ki67 index (**B**,**C**) preoperatively (preop., 0 h), and at 24 h, 48 h, 72 h, and 168 h after PVL and ALPPS (*n* = 6 per time point per group). * *p* < 0.050, ** *p* < 0.0010 versus PVL; ## *p* < 0.001 PVL versus corresponding controls (preop.); §§ *p* < 0.0010 ALPPS versus corresponding controls (preop.). Statistical analysis was performed with a two-way ANOVA and Tukey’s post hoc test. Data are presented as mean ± 1 standard deviation (SD).

Both PVL and ALPPS induced growth of the right part of the median lobe from 24 h to 168 h (Figure 3A), whereby an increase in liver mass of the non-ligated lobes was higher in the ALPPS group compared to the PVL group at all time points. The degree of atrophy in the ligated lobes (right lateral, left lateral, caudate, and left part of the median lobe) increased over time in the PVL and ALPPS group, confirming that the interventions had been properly implemented (Supplementary Figure S1). Cell proliferation in the non-ligated lobes in both groups was higher from 24 h to 72 h versus baseline and abated towards baseline levels at the end of the experiment (Figure 3B,C). The Ki67 index in the right part of the median lobe was higher in the ALPPS group compared to the PVL group at 24 h and 48 h. For histological alterations in the non-ligated lobe see Supplementary Figure S2.

### 3.2. Lobular Microcirculation Decreases after ALPPS in the Ligated Lobes

**Figure 4:**
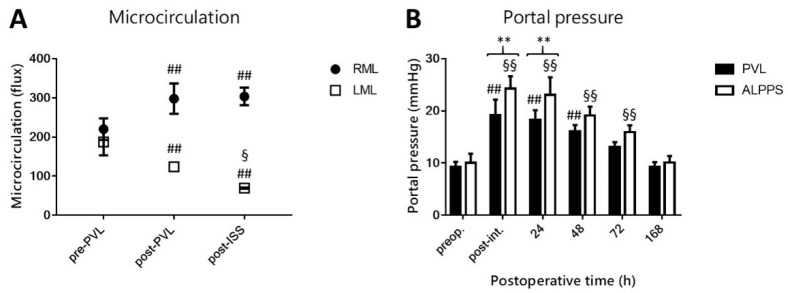
Effect of portal vein ligation (PVL) and associating liver partition and portal vein ligation for staged hepatectomy (ALPPS) on the liver microcirculation and portal pressure. The microcirculatory flow measurements (**A**) were performed preoperatively (pre-PVL), after PVL (post-PVL), and after the in-situ split in ALPPS (post-ISS) in the non-ligated right median lobe (RML) and the ligated left median lobe (LML) (*n* = 6 per time point per group). ## *p* < 0.001 versus preoperative value (pre-PVL); § *p* < 0.05, §§ *p* < 0.001 versus post-PVL value (post-PVL). The portal pressure measurements (**B**) were performed preoperatively (preop.), immediately after intervention (post-int.) and at 24 h, 48 h, 72 h, and 168 h after PVL and ALPPS (*n* = 6 per time point per group). ** *p* < 0.001 versus PVL; ## *p* < 0.001 PVL versus corresponding controls (preop.); § *p* < 0.05, §§ *p* < 0.001 ALPPS versus corresponding controls (preop.). Statistical analysis was performed with a two-way ANOVA and Tukey’s post hoc test. Data are presented as mean ± 1 standard deviation (SD).

Contrived occlusion of blood supply to portions of the liver causes a divergence of blood flow between the ligated and the non-ligated liver segments. In line with this, both PVL and ALPPS resulted in an increase in microcirculatory flow in the right median lobe while flow in the ligated left median lobe had decreased compared to the pre-ligation flow (Figure 4A). The transection part of ALPPS exerted no additional effect on flow in the non-ligated right median lobe but further decreased flow in the ligated left median lobe.

### 3.3. Increased Portal Pressure following ALPPS

Both interventions were associated with an increase in portal pressure immediately and at 24 h post-intervention, after which the pressure gradually normalized by the end of the experiment (Figure 4B). These findings are consistent with the fact that an equal amount of blood is directed into the smaller liver volume after PVL and support the microvascular flow data. Compared to baseline, the portal pressure remained elevated for 48 h in the PVL group and for 72 h in the ALPPS group. At 168 h after the intervention the portal pressure had reverted to baseline level. The increase in portal pressure was greater in the ALPPS group compared to the PVL group during the first 24 h.

### 3.4. Increased Systemic and Portal Bile Acid Concentration following ALPPS

**Figure 5:**
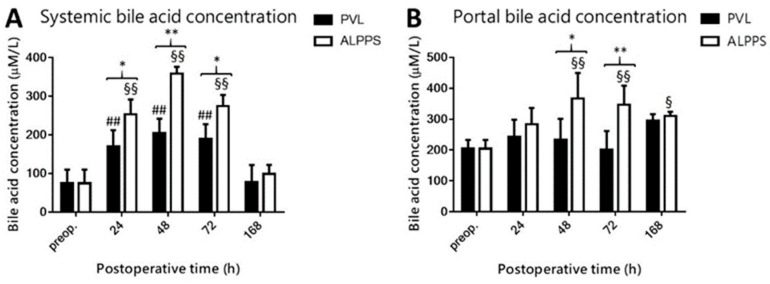
Effect of portal vein ligation (PVL) and associating liver partition and portal vein ligation for staged hepatectomy (ALPPS) on systemic bile acid (BA) concentration (**A**) and portal BA concentration (**B**). BA levels were determined preoperatively (preop.) and at 24 h, 48 h, 72 h, and 168 h after PVL and ALPPS (*n* = 6 per time point per group). * *p* < 0.05, ** *p* < 0.001 versus PVL; ## *p* < 0.001 PVL versus corresponding controls (preop.); § *p* < 0.05, §§ *p* < 0.001 ALPPS versus corresponding controls (preop.). Statistical analysis was performed with a two-way ANOVA and Tukey’s post hoc test. Data are presented as mean ± 1 standard deviation (SD).

PVL and the ALPPS resulted in a spike in systemic BA concentration from 24 h to 72 h after the intervention, which normalized to baseline levels at 168 h (Figure 5A). Systemic BA concentration was higher in the ALPPS group compared to the PVL group from 24 h to 72 h. BA levels in the portal circulation did not change in the PVL group but became elevated from 48 h to 168 h following ALPPS, which resulted in a disparity between the groups from 48 h to 72 h (Figure 5B).

### 3.5. Changes in the Expression of Bile Acid Transporters and Production Enzymes Following ALPPS

**Figure 6:**
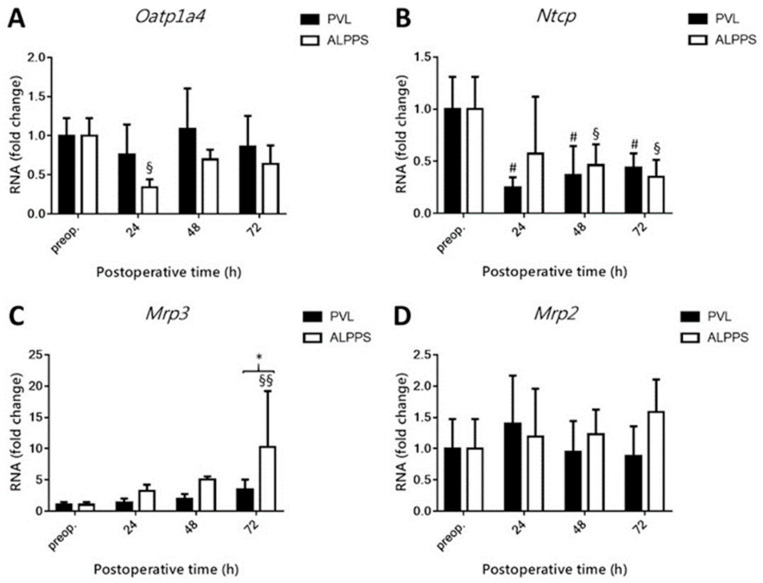

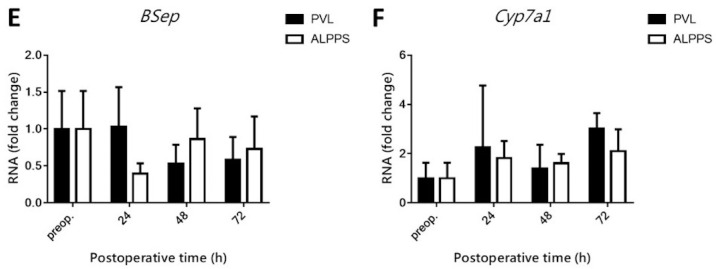
Effect of portal vein ligation (PVL) and associating liver partition and portal vein ligation for staged hepatectomy (ALPPS) on mRNA levels of hepatic bile acid production enzymes and transporters. Transcript levels of Oatp1a4 (**A**), Ntcp (**B**), Mrp3 (**C**), Mrp2 (**D**), Bsep (**E**), and Cyp7a1 (**F**) were determined preoperatively (preop.) and at 24 h, 48 h, 72 h, and 168 h after PVL and ALPPS (*n* = 6 per time point per group). * *p* < 0.05, # *p* < 0.05 PVL versus corresponding controls (preop.); § *p* < 0.05, §§ *p* < 0.001 ALPPS versus corresponding controls (preop.). Statistical analysis was performed with a two-way ANOVA and Tukey’s post hoc test. Data are presented as mean ± 1 standard deviation (SD).

Following PVL, hepatocytes in the right median lobe reduced the expression of the BA importer *sodium taurocholate co-transporting polypeptide (Ntcp)* in the overall basolateral transport cascade, with no changes in *organic anion transporting polypeptide (Oatp)1a4* and *multidrug resistance protein (Mrp)3* levels (Figure 6A–C). ALPPS, in contrast, accounted for a reduction in *Oatp1a4* (at 24 h post-intervention; Figure 6A) and *Ntcp* (at 48 h and 72 h post-intervention; Figure 6B), which suggests an attempt towards reaching BA homeostasis following a surge in BA influx. This is supported by the increase in transcript levels of the basolateral exporter *Mrp3* at 72 h post-ALPPS (Figure 6C). Neither intervention had a notable effect on canalicular export of BAs via *Mrp2* or *bile salt exporting pump (Bsep)* (Figure 6D, E, respectively) or BA synthesis, as reflected by the unchanged expression levels of *cytochrome P450 isoform 7A1 (Cyp7a1)* (Figure 6F).

### 3.6. Hepatic Fxr Signaling Is Downmodulated after PVL and ALPPS

**Figure 7:**
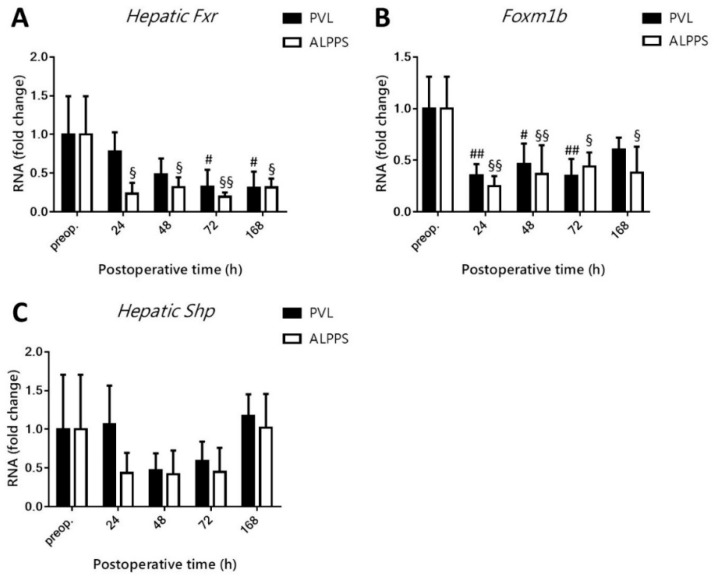
Effect of portal vein ligation (PVL) and associating liver partition and portal vein ligation for staged hepatectomy (ALPPS) on the hepatic Fxr pathway. Hepatic Fxr (**A**), Shp (**B**), and Foxm1b (**C**) transcript levels were determined preoperatively (preop.) and at 24 h, 48 h, 72 h, and 168 h after PVL and ALPPS (*n* = 6 per time point per group). # *p* < 0.05, ## *p* < 0.001 PVL versus corresponding controls (preop.); § *p* < 0.05, §§ *p* < 0.001 ALPPS versus corresponding controls (preop.). Statistical analysis was performed with a two-way ANOVA and Tukey’s post hoc test. Data are presented as mean ± 1 standard deviation (SD).

As presented in Figure 7A, hepatic *Fxr* levels decreased after PVL in the hypertrophy response phase (72 h to 168 h). ALPPS also caused a decline in *Fxr* levels, but earlier than 24 h, and remained lower relative to preoperative levels up to 168 h after the intervention. No intergroup differences were observed at any of the time points. *Foxm1b*, the downstream target of *Fxr*, exhibited a similar trend to *Fxr* in the PVL-exposed animals in that its transcript levels were decreased compared to baseline (Figure 7B). In the ALPPS group *Foxm1b* levels were also lower relative to preoperative levels at all time points, altogether suggesting anti-mitogenic signaling in this pathway, which is in contrast to the liver growth data in Figure 1. *Shp* did not seem to interfere in BA signaling following PVL and ALPPS given the absence of dysregulation (Figure 7C).

### 3.7. ALPPS Induces Upregulation of Ileal Fxr and Fgf15 and Hepatic Fgfr4 mRNA Levels

**Figure 8:**
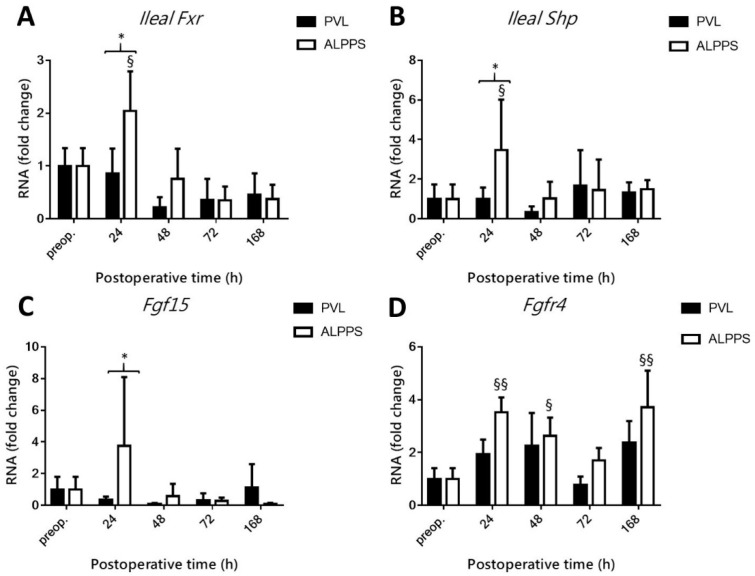
Effect of portal vein ligation (PVL) and associating liver partition and portal vein ligation for staged hepatectomy (ALPPS) on the intestinal Fxr pathway. Intestinal Fxr (**A**), intestinal Shp (**B**), Fgf15 (**C**), and Fgfr4 (**D**) were determined preoperatively (preop.) and at 24 h, 48 h, 72 h, and 168 h after PVL and ALPPS (*n* = 6 per time point per group). * *p* < 0.05; § *p* < 0.05, §§ *p* < 0.001 ALPPS versus corresponding controls (preop.). Statistical analysis was performed with a two-way ANOVA and Tukey’s post hoc test. Data are presented as mean ± 1 standard deviation (SD).

Ileal *Fxr* showed no change after PVL but exhibited a spike at 24 h after ALPPS, while remaining at similar levels to baseline at all other time points in both groups (Figure 8A). This resulted in a notable difference between ALPPS and PVL at 24 h. Concomitantly, *Shp* had remained unchanged in the PVL group, while its upregulation was observed in the ileum of ALPPS-subjected animals at 24 h post-intervention, which had receded to baseline levels at the subsequent time point (Figure 8B). Ileal *Fgf15* coincidentally did not change in the PVL group. A single time point spike was observed after ALPPS at 24 h (Figure 8C). Upregulation of *Fgfr4* was not observed after PVL. ALPPS-exposed hepatocytes had upregulated *Fgfr4* at 24 h, 48 h, and 168 h (Figure 8D), the protein product of which constitutes the cognate receptor for ileal Fgf15.

## 4. Discussion

ALPPS is an emerging surgical intervention offered to select patients with liver tumors that are inoperable by standard techniques. As with PVL, which comprises an integral part of ALPPS, the main aim is to amplify the rate of liver regeneration in the FLR in order to enable safe resection of the tumor-containing liver lobes. As the activation of primary nuclear BA receptor Fxr has been identified as an important mediator of liver regeneration [9,21], we investigated the role of BAs and the comprehensive *Fxr* signaling axis (hepatic and ileal) in the context of liver regeneration following PVL and ALPPS. At the time of this study, the ALPPS-related information was missing from the existing literature. Adding this to the knowledge pool was deemed important insofar as ALPPS outcomes may be further improved by pharmacological intervention using BA analogues [16,17,22].

The main findings of the study were that [1] ALPPS induced more liver growth in the non-ligated lobe than PVL; [2] the BA concentration in the systemic and portal circulation was more profoundly elevated after ALPPS than after PVL; [3] although hepatocytes seemed to take up BAs from the circulation, the BAs did not activate Fxr signaling following either intervention; and [4] ALPPS, but not PVL, induced transient transcriptional upregulation of ileal *Fxr* and *Fgf15*, which coincided with an increase in hepatic *Fgfr4* expression levels. Accordingly, we concluded that the surgically-induced liver growth following ALPPS was likely mediated by the ileal Fxr-Fgf15 signaling axis, whereby on the hepatic end the Fgfr4 downstream signaling presumably proceeded via routes other than Fxr-Foxm1b. These findings refute our hypothesis regarding BA-mediated hepatocellular proliferation but confirm the ileal signaling aspects.

Since our results are partly in contrast to the putative framework of BA-mediated liver regeneration following a surgical intervention such as hepatectomy [8], there is a need to validate the animal model used in the current experiments and assess the possibility that technical elements rather than physiological and biochemical phenomena accounted for the deviation from expected results. Accordingly, pertinent data were benchmarked against reported findings by others. Firstly, we observed more extensive liver growth in the ALPPS group compared to the PVL group, which is in accordance with the experimental and clinical literature [6,20,23,24,25]. The increase in the Ki67 index shows efficient hepatocyte proliferation in the non-ligated lobe, indicating that the liver growth is not due to cellular swelling, which is also supported by previous findings [26]. Secondly, PVL and ALPPS considerably affect the circulation of the liver [27,28], temporarily impairing the microcirculation in the ligated lobes [24,27] and elevating portal pressure as the venous blood coming from the splanchnic circulation has to pass through a decreased vascular diameter [29,30]. Using LDF and portal pressure measurements it was confirmed that our model yielded the same results for both interventions as reported by others. Microvascular flow was more significantly impaired in the left median lobe after transection, which may be attributable to the disruption of collateral circulation by the transection that is known to compound differences in portal flow between the ligated and non-ligated lobes [31]. Moreover, intestinal permeability linked to portal hypertension activates intestinal Fxr signaling [32], which is even more pronounced in case of liver injury [33], and therefore more applicable to ALPPS than to PVL. Our findings, which related to ileal Fxr following PVL (no upregulation) versus ALPPS (upregulation at 24 h after intervention), are consistent with the literature. Thirdly, BA plasma levels increase rapidly after partial hepatectomy as the uptake capacity is diminished after partial removal of the tissue responsible for BA plasma clearance [11,12,34,35]. Moreover, regeneration of the canalicular networks in the FLR are poorer following ALPPS than after portal vein embolization, which leads to leakage of BAs into the bloodstream [36]. These phenomena are echoed in our results if one accepts the premise that PVL mimics partial hepatectomy with respect to the access of hepatocytes to the circulatory BA pool and that PVL resembles portal vein embolization in terms of the occlusion of blood flow to the dedicated hepatic regions. Correspondingly, the elevation of plasma BA levels was observed after PVL and ALPPS in our model, whereby ALPPS resulted in higher BA levels than PVL. The synchrony between our model and the literature therefore dismisses the likelihood that our deviating findings concerning the molecular signaling and biological responses were rooted in technical flaws.

The most pressing questions therefore are: [1] “why does the FLR after ALPPS ignore BA-derived mitogenic signals?” and [2] “how does ALPPS induce liver hypertrophy without engaging the BA-driven Fxr-Foxm1b pathway?” To address the first question, it has been well-established that rat livers express hepatic transporters that mediate BA influx after a liver-reducing procedure such as partial hepatectomy [17,22,37,38], which results in their transcriptional regulation [17,22] to balance hepatocellular BA loading and BA composition [22,39]. A volume-restrictive intervention also results in hepatic Fxr signaling [17,22], which in our model was clearly downmodulated after PVL and subsequent to the transection of the median lobe. Given the elevated BA concentration in the systemic and portal circulation, we first investigated whether hepatocytes were responsive to the increased circulatory BA supply after PVL and ALPPS. BAs are imported into hepatocytes at the basolateral end by OATPs and NTCPs, and exported basolaterally by MRP3 and 4 as well as OSTα and β. At the canalicular end, BAs are exported into the biliary tract by MRP2, BSEP, and by multidrug resistance-associated protein (MDR)2. Instead of importing BAs, hepatocytes can also produce BAs from cholesterol, which is mediated by CYP7A1 [8].

Although not measured, we believe that hepatocellular BA loading likely occurred because of the elevated BA levels in the systemic and portal circulation, and because hepatocytes responded by downregulating transcript levels of both basolateral importers and by increasing mRNA expression of the basolateral exporter Mrp3. Mrp3 is regulated independently of Fxr [40,41] and functions to curtail excessive exposure to toxic BAs [41]. This suggests that hepatocytes were attempting to reduce intracellular BA levels by enforcing basolateral modifications. Why no canalicular measures were implemented after ALPPS is unclear, but it is possible that the basolateral course of action was sufficient to restore hepatocellular BA homeostasis. It is equally unclear how, if indeed BA loading occurred after ALPPS, the BAs were prevented from activating the Fxr pathway. Further speculation is futile without information on the composition of hepatocellular BAs after ALPPS or molecular intervention studies in the Fxr-Foxm1b pathway.

With respect to the second question, liver regeneration can occur in the absence of BA signaling. For example, and in the context of our observations, ileum-derived Fgf15 is a potent trigger of hepatocellular proliferation via the Fgfr4 receptor on hepatocytes. Upon binding, Fgfr4 can signal mitosis independently of Foxm1b via mitogen activated protein kinases (MAPK) and via the nuclear factor kappa-light-chain-enhancer of activated B-cells (NF-κB). Activation of the Stat3 pathway by Fgf15 binding to Fgfr4 [42] has been observed following ALPPS [43,44], but did not play a role in our model given the downregulation of *Foxm1b*, a downstream target of Stat3 [8]. Alternatively, BAs can bind to the nuclear receptor pregnane X receptor (PXR) and trigger mitosis while bypassing Stat3 and Foxm1b. These pathways have been elaborated in [8] and constitute plausible explanations for the ALPPS-related observations. Lastly, it should be noted that BAs are only one class in a plethora of complete mitogens and auxiliary mitogens that steer surgery-induced liver regeneration [8]. Reducing or eliminating BAs from the equation does not abrogate liver regeneration but only stalls it [9,21,45]. Similarly, liver-specific Fxr-null mice undergo stalled liver regeneration compared to wild-type animals [9,14].

In the final analysis, ALPPS-induced liver hypertrophy is mediated by factors other than hepatic Fxr, which likely entails the Fgf15/Fgfr4 signaling axis. This does not mean that pharmacological interventions centered on BA metabolism are therapeutically moot. On the contrary, we have demonstrated here that the starting point of liver hypertrophy is ileal Fxr. Accordingly, through combining ALPPS with orally administered obeticholic acid, a synthetic mitogenic BA analogue that amplifies liver regeneration via Fxr [17] after radiological interventions such as portal vein embolization [16], there seems to be a combinatorial modality that warrants exploration.

It is important that readers contextualize our study in light of several limitations. Firstly, the molecular analyses were performed at the transcriptional level. The data should be confirmed at the proteomic level. Secondly, no measurements were performed on intrahepatic BA concentrations and on the composition of the hepatocellular BA pool, which would have provided additional insight into the relationship between ALPPS, BAs, and hepatocyte proliferation. Thirdly, no molecular intervention studies were performed, such as siRNA-mediated *Fxr* knockdown, antibody neutralization of circulatory Fgf15 or blockade of Fgfr4, or biliary drainage to remove BAs from the intestines.

In conclusion, our study revealed key physiological processes that could underlie BA-induced liver regeneration following ALPPS. Based on our results, BA-activated mitotic signals could be characterized by the activation of the intestinal Fxr pathway rather than hepatic Fxr signaling. While the hepatic Fxr pathway occupies a central role in post-hepatectomy liver regeneration [10,14,21], our findings point to a different pattern of BA-induced liver regeneration following ALPPS. Although the underlying mechanisms require further clarification, BAs drive liver regeneration following ALPPS and contribute to improved FLR volume, and possibly survival, after second-stage extended hepatectomy. We therefore propose that intestinal Fxr should be investigated as a potential therapeutic target to enhance post-operative outcomes following ALPPS, which can be achieved through mitogenic BA mimetics that have been cleared by regulatory agencies and are being clinically implemented for other indications.

## Data Availability

The data presented in this study are available upon request to the corresponding author.

## Supplementary Materials

The following are available online at https://www.mdpi.com/article/10.3390/curroncol28060438/s1, Figure S1: Rate of atrophy, Figure S2: Histological structure of liver preoperatively and after the operation, Table S1: qRT-PCR primers.
