# Supplementary material for: The Role of Farnesoid X Receptor in Accelerated Liver Regeneration in Rats Subjected to ALPPS
Source: Curr Oncol. 2021 Dec 9;28(6):5240–54. doi: 10.3390/curroncol28060438 (PMC8700148; doi:10.3390/curroncol28060438)
Supplement: Supplementary file 1 [file curroncol-28-00438-s001.zip › curroncol-1488854-supplementary.pdf]

Current Oncology

The Role of Farnesoid X Receptor in Accelerated Liver Regeneration in Rats Subjected to ALPPS

Noemi Daradics, Pim B. Olthof, Andras Budai, Michal Heger, Thomas M. van Gulik, Andras Fulop and Attila Szijarto

Table S1. qRT-PCR primers

Target	Forward (5′ → 3′)	Reverse (5′ → 3′)
Cyp7a1 (RML)	GCAGCCTCTGAAGAAGTGAG-TGG	GATGCTGTCTAGTACCGGCAGG
Ntcp (RML)	TGAACCTCAG-CATCGTGATGACC	GGACGATCCCTATGGTGCAAGG
Mrp3 (RML)	ATGCTGGCCAAAATGCGGTTGC	CCAGGAGCCCTTGCAGTATTCC
Bsep (RML)	CTCTGCTTTGCCTTTTCCCAGG	AGAGACCACCCTGAAAACGTGG
Mrp2 (RML)	GAGTCTGAG-GATGAATCTCGACC	TGCCCTATGCTCAGGTTGTCACC
Fxr (RML; I)	GTCATCCTCTCTCCAGACAG-ACA	GGTTGAATGTCCGGAGTTCTGTC
Shp (RML; I)	GCTAGAGGAACCCAACAGTGGT	CCTGGCACATCTGGGTTGAAGA
B2m (RML)	CCACCGGAGAATGGGAAGCCC	TCTCGGTCCCAGGTGACGGT
Foxm1 (RML)	AGGCGCCCTCAAGAGCATCA	TGGTGCCAACACTTCCAGCCT
Fgfr4 (RML)	AGCTCCAGGCGGGTGAGTGT	CGCTGACCACCTTCCTGGCT
Fgf15 (I)	AGGGCCAGAAACCTTCAAAC	GATCCATGCTGTCGCTCTC
Oatp1a4 (RML)	CTCTTATGAAGGGGTTCAG-CACC	CAAGGCAGGCTGACATGTATGC

RML: right median lobe of the liver, I: identical part of the ileal tract.

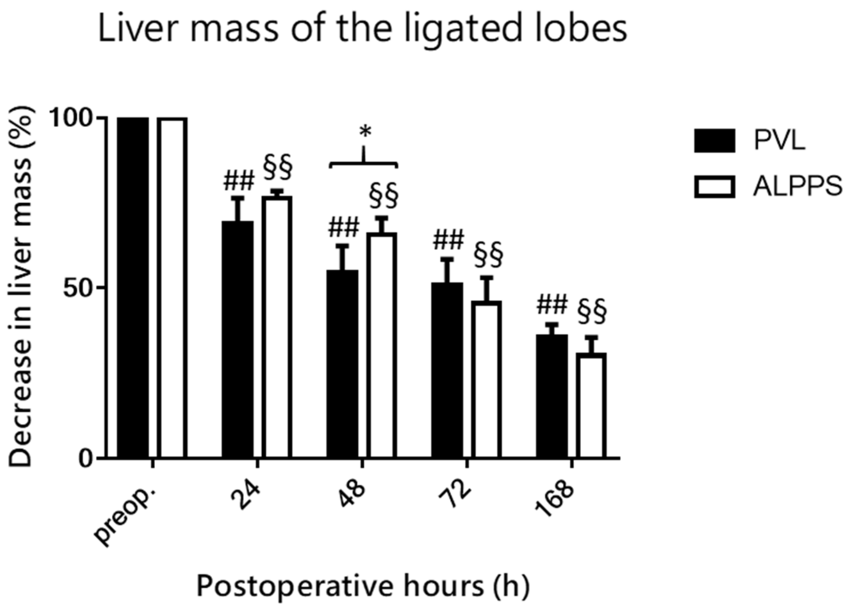

Figure S1. Rate of atrophy. Decrease in liver mass of the atrophying liver lobes preoperatively (preop.) and at 24 h, 48 h, 72 h, and 168 h after portal vein ligation (PVL) versus associating liver partition and portal vein ligation for staged hepatectomy (ALPPS) (N = 6 per time point per group).

* $P < 0.05$ versus PVL; ## $P < 0.001$ PVL versus corresponding controls (preop.); §§ $P < 0.0010$ ALPPS versus corresponding controls (preop.). Statistical analysis was performed with a two-way ANOVA and Tukey's post hoc test.

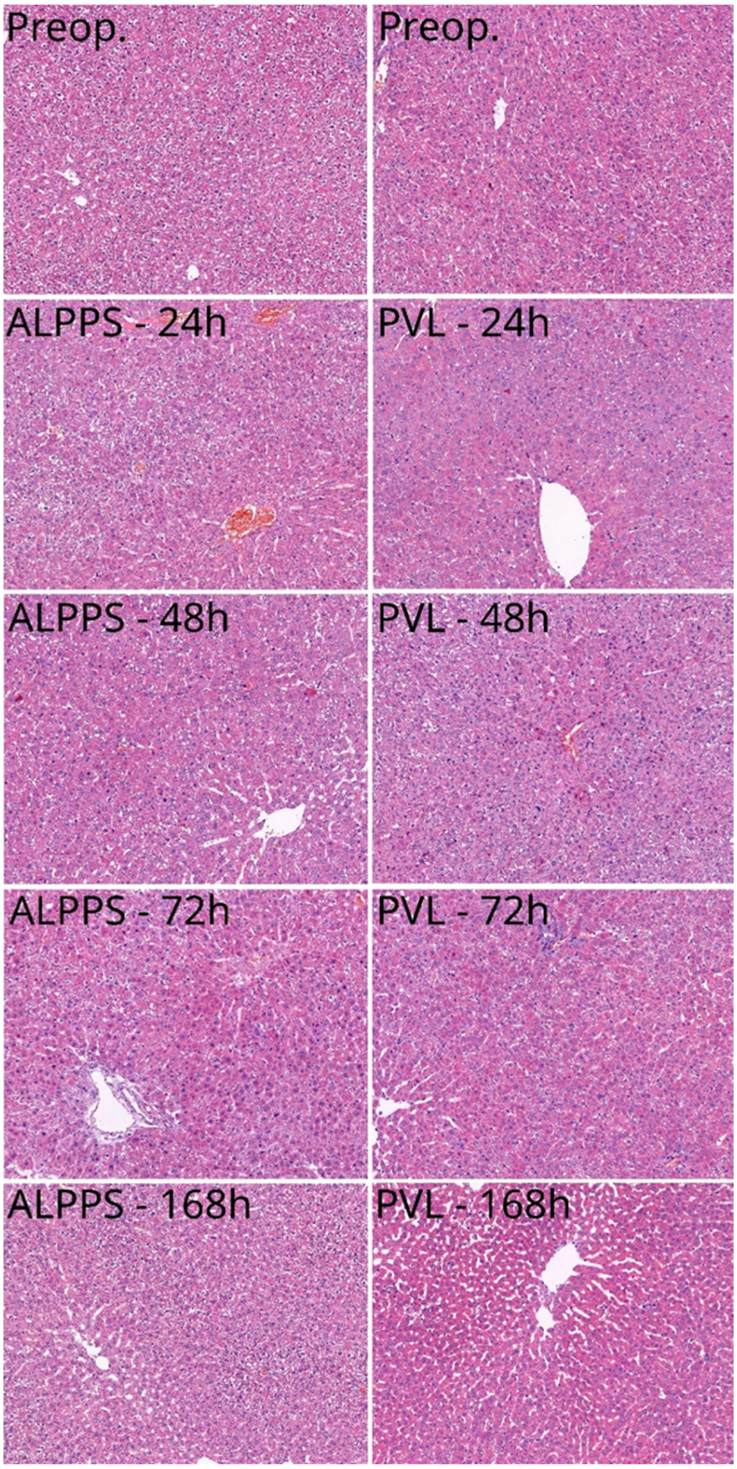

Figure S2. Histological structure of liver preoperatively and after the operation. Histological structure (hematoxylin and eosin stain; original magnification: ×150) of the regenerating right median (RM) lobe preoperatively (preop.) and at 24 h, 48 h, 72 h, and 168 h after associating liver partition and portal vein ligation for staged hepatectomy (ALPPS) and portal vein ligation (PVL).
